# Assessment of healthcare application quality: Development of a standardized methods for healthcare professionals

**DOI:** 10.1016/j.mex.2023.102391

**Published:** 2023-09-27

**Authors:** Desirèe Andreoli, Stefano Mancin, Marco Sguanci, Mattia Ricco, Domenica Gazineo, Lea Godino

**Affiliations:** a Azienda Ospedaliera Santa Maria della Misericordia, Perugia, Italy; b SIAN, Società Infermieri Area Nefrologica, Olbia, Italy; c Department of Biomedicine and Prevention, University of Rome “Tor Vergata”, Rome, Italy; d Department of Medicine and Surgery, Research Unit of Nursing Science, Università Campus Bio-Medico di Roma, Italy; e Department of Medical and Surgical Sciences (DIMEC), University of Bologna, Bologna, Italy; f Governo Clinico e Qualità, IRCCS Azienda Ospedaliero-Universitaria di Bologna, Bologna, Italy; g Medical Genetics Unit, IRCCS Azienda Ospedaliero-Universitaria di Bologna, Bologna, Italy

**Keywords:** Chronic kidney disease, Nutrition, Mobile apps, Mobile-health technology, Assessment of health mobile apps quality

## Abstract

The advancement of the mobile app market is reshaping healthcare, emphasizing the imperative for quality and efficacy in health applications. This methodology has been devised to assess mobile health applications, aiming to assist healthcare professionals in selecting apps for e-healthcare consumers. Key facets of this approach are:

• A stringent selection process within mobile app stores
• A standardized assessment using the Mobile App Rating Scale to achieve consistent and replicable evaluations, systematically organizing app evaluations
• A comprehensive framework guiding healthcare practitioners in determining which apps to integrate into clinical practice and which to exclude

Central to this method is the emphasis on distinguishing apps that enhance clinical practice from those that fall short in important areas such as the effectiveness of proposed health features, data accuracy, adherence to clinical guidelines, data security, and user privacy. With heightened attention to usability and accessibility, the methodology also addresses the common risks inherent in mHealth implementation, ensuring that selected apps not only meet technical criteria but also align with the broader healthcare ecosystem's needs and challenges.

## Graphical abstract

**Figure:**
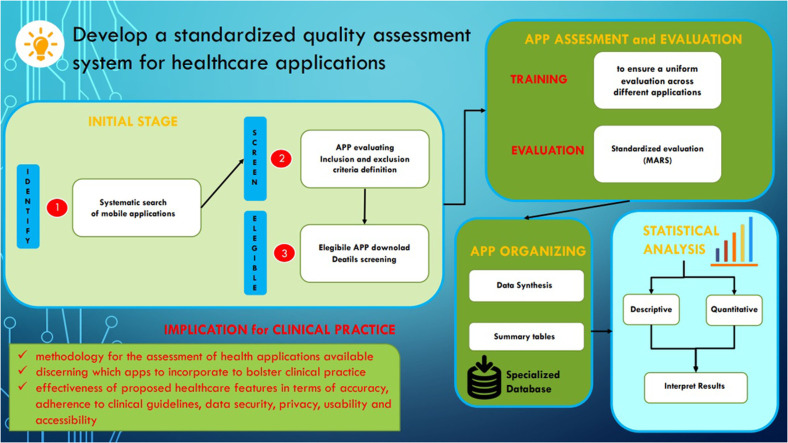

Specifications table

**Table:** 

Subject area:	Medicine and dentistry
More specific subject area:	*Mobile health apps*
Name of your method:	Assessment of health mobile apps quality
Name and reference of original method:	*Mobile app rating scale: a new tool for assessing the quality of health mobile apps.*
Resource availability:	doi:10.2196/mhealth.3422.

## Introduction

The technological advancement of recent decades has had a profound impact on multiple sectors of daily and professional life, and healthcare is no exception [1]. In particular, the mobile applications market has experienced exponential growth, becoming an ubiquitous tool for accessing information, entertainment, work, and, last but not least, health [2]. The proliferation of applications was made possible thanks to the creation of app stores, digital platforms dedicated to distributing applications for mobile devices [3]. The two most important app stores worldwide are the Apple App Store, for iOS devices, and the Google Play Store, for Android devices. These two giants hold the majority of the market, with millions of applications available for download [4]. The Apple App Store, launched in 2008, inaugurated the era of mobile applications, becoming the reference point for iOS device users [5]. The Google Play Store, formerly known as Android Market, is the main application store for the Android operating system, hosting a wide range of applications, from games to utilities, from educational to health apps [6].

The presence of these platforms has made the publication and distribution of applications a simpler and more accessible process for developers, leading to an exponential increase in the number of available applications [7]. This increase has opened up new opportunities but has also posed a series of challenges, above all in the health sector and, especially towards acute and chronic diseases with a high impact on global health systems [8], [9], [10], [11], [12], where the quality and accuracy of the information provided are of vital importance [13]. The manufacturers of these applications are varied: from companies specializing in medical technology to scientific societies, to individual developers or independent development teams. This variety of creators is undoubtedly a source of innovation but also brings with it a series of potential issues [14].

A critical aspect of this proliferation of healthcare apps concerns their quality and effectiveness. Not all applications undergo rigorous quality control or scientific evaluation before being made available to the public [15]. This can lead to situations where apps do not provide the promised service, or worse, provide incorrect or misleading information [16]. In addition, privacy and data security issues may arise, given the sensitive nature of the health information managed by these applications [17]. An inadequate evaluation of healthcare applications can lead to a series of negative consequences, including the spread of inaccurate medical information, non-compliance with clinical guidelines, violation of patient privacy, and a general lack of transparency [18]. As a result, the choice of healthcare applications by health professionals to recommend to their patients can become a complex and potentially risky process [19].

### Research protocol objective

The objective of this research methods is to develop a standardized quality assessment system for healthcare applications. This method is designed to be used by teams of healthcare professionals or research groups, rather than by single individuals, in order to ensure a thorough and objective analysis. The application of the method by a team allows for a more balanced evaluation, minimizing potential biases that could emerge if the method were applied by a single professional. The method covers various key aspects: the effectiveness of the proposed healthcare features, the accuracy of the information provided, compliance with clinical guidelines, data security and privacy, and finally the usability and accessibility of the user interface.

Furthermore, this method is designed to be flexible and able to adapt to the new challenges and changes characterizing the rapid and constant evolution of the application market. This work represents a significant step forward in ensuring that the potential offered by mobile technology is best exploited to improve patient health and wellbeing, while minimizing associated risks.

## Methods

### Mobile app selection and evaluation

#### Search strategy

In a preliminary phase, a thorough analysis of relevant documents and clinical practice guidelines released by recognized health organizations and foundations should be carried out. This initial step is crucial to gain an adequate understanding of current best practices and official recommendations in the study area. This information can also aid in defining the inclusion and exclusion criteria for the apps to be evaluated. Subsequently, a comprehensive and systematic search of mobile applications will be conducted by two academic researchers to ensure objectivity and reduce the risk of bias. The main databases for this search should be Google Play Store and Apple Store, given their predominance in the mobile application market. The use of two researchers will allow for a comparison and contrast of results, thus enhancing the reliability of the selection process.

As the use of abbreviations and logical operators is not possible in the app store databases, each relevant keyword for the study area should be entered individually. This method will increase the likelihood of identifying all pertinent applications. In addition to the systematic search, a manual search using Google or other search engines should be conducted. This additional step can help to identify any application that might have been missed during the systematic search.

#### Selection of mobile apps and inclusion and exclusion criteria

When planning a comprehensive assessment of mobile applications for health purposes, the research will be conducted by two independent academic researchers. The decision to involve two reviewers not only adds to the robustness of the methodology by reducing the potential for bias, but also contributes to the overall reliability of the assessment, given that different reviewers might perceive and interpret the same app features differently. The search process itself needs to be thorough and meticulously conducted. It would involve the identification of the apps, a preliminary evaluation based on the app store descriptions, and the subsequent download of the chosen applications for a more detailed screening.

Establishing clear inclusion criteria from the outset would be crucial. Some potential criteria might include: (1) the language in which the application is offered, taking into account the linguistic and cultural context of the target population for the study, (2) the demographic or population that the application is designed to serve, ensuring a match with the intended study group, (3) the specific health condition addressed by the application, ensuring direct relevance to the subject of study, (4) the presence of personalized healthcare programs within the application, indicative of the level of user-centered approach of the app, (5) the cost factor, favoring free applications or those offering a trial period of at least 14 days, to ensure equitable accessibility and usability for a diverse range of users, and (6) the privacy policy, selecting the apps that guarantee data protection and respect for user privacy.

In the same vein, well-defined exclusion criteria should also be set. For instance, one might exclude mobile health apps focusing on health conditions that are outside the research scope, to ensure that the selected apps are all pertinent and related directly to the research interest. Furthermore, apps created specifically for professional healthcare providers could also be excluded as they may introduce a level of complexity not intended for the general public, and could therefore skew the study results.

#### Selection and detailed description of a standardized rating scale for mobile apps evaluation

Several tools are available for assessing the quality of health mobile apps. In 2023, Jacob et all. conducted a comprehensive review of eHealth tools, resulting in the categorization of 36 unique criteria into 13 clusters [20]. These criteria cover technical, social, and organizational aspects of assessment. Technical criteria include aspects, functionality, content, data management, and design, while social criteria encompass human centrality, health outcomes, visible popularity metrics, and social aspects. Organizational criteria cover sustainability, scalability, healthcare organization, context, and developer considerations. Despite the availability of numerous tools, there lacks a standardized framework for the comprehensive assessment of electronic healthcare tools. Instead, multiple frameworks exist, each with its curated set of tools, designed by various authors sharing the common objective of furnishing a valid assessment tool for mHealth applications.

In this context, we believe that the ideal scale should possess key attributes to ensure a precise and valuable assessment of applications, namely:

• comprising a toolkit that encompasses fundamental dimensions, such as information accuracy and reliability, usability, accessibility, data security, regulatory compliance, interactivity, and engagement;
• demonstrating reliability, objectivity, and impartiality through subsequent validation studies;
• featuring ease and expediency of use;
• utilizing quality assessment criteria derived from a systematic analysis and synthesis of pertinent literature.

In the landscape of assessing the quality of mHealth apps several evaluation tools have emerged. These include Enlight [21], the assessment matrix proposed by Khoja et al. [22], the Health Care Information and Management Systems Society (HIMSS) guidelines [23], and the Mobile App Rating Scale (MARS) [24].

Our perspective underscores the suitability of the MARS scale for a methodical and standardized evaluation of the selected mobile apps. Originally developed by a team of researchers in Australia, the MARS provides a comprehensive multidimensional measure for the classification and assessment of mobile health apps, making it an invaluable tool in this context (Supplementary File).

The MARS scale consists of 19 items divided into four broad sections, each assessing a different aspect of the app's quality. The “Engagement” section, for example, evaluates the app's ability to capture and sustain user interest over time. This is an essential feature of any app as sustained engagement contributes significantly to the app's efficacy. In this section, factors such as the entertainment value, the degree of customization allowed by the app, and the extent of interactivity are considered.

Next, the “Functionality” section focuses on the app's performance and user-friendliness. It examines the ease of use, the navigation structure, and the gestural design of the app. These are all critical factors that can directly affect the user's experience and ultimately influence the app's effectiveness.

The “Aesthetics” section appraises the visual aspects of the app. It takes into consideration the graphic designs, overall esthetic appeal, color schemes, and the style consistency. Although seemingly trivial, these factors can significantly impact the user's perception of the app and their willingness to use it regularly. Lastly, the “Information” section assesses the app's content in terms of its credibility, relevance, and quality. This section is particularly crucial for health-related apps, as the reliability of the information provided can directly impact the health outcomes of the users. It scrutinizes various elements such as the accuracy of app descriptions, goals, quality and quantity of information provided, visual information, credibility of the sources of information, and evidence base. In addition to these four main sections, MARS includes a “Subjective Quality” section, giving an overall user impression and a “Specificity” section that assesses the app's potential to change the user's knowledge, attitudes, and intentions to change behavior.

The MARS scale is available in various languages including, but not limited to, Spanish (MARS-S) [25], Italian (MARS-I) [26], German (MARS-G) [27], and French (MARS-F) [28]. This makes the scale a versatile and adaptable tool in diverse international research contexts.

The MARS scale distinguishes itself from other assessment instruments by its rigorous foundation in a systematic analysis and synthesis of pertinent literature. Unlike several other scales, the MARS scale doesn't arbitrarily select criteria but ensures their relevance through a thorough literature-based approach [29]. This feature enhances its credibility as a tool for assessing app quality.

Furthermore, the MARS scale offers practical advantages. Its user-friendliness and efficiency stand out, as it requires only a brief 15-minute preparation by professionals. This is a marked contrast to more intricate and time-consuming matrices like that of Khoja et al. [22].

The Health Information Management Systems Society (HIMSS) guidelines, while valuable for assessing app usability in terms of efficiency, effectiveness, and user satisfaction, neglect the criteria for evaluating the quality of the information. This omission is critical, as inaccuracies or inadequacies in healthcare information within mHealth apps can jeopardize user safety. In contrast, the MARS scale comprehensively addresses information quality as one of its fundamental criteria.

Regarding Enlight [21], while it boasts a comprehensive set of items, its recent development renders it empirical, necessitating further research to establish its validity in predicting the effectiveness of electronic healthcare programs. It looks like the main corrections were “The Health Information Management Systems Society” at the beginning and “Regarding Enlight” later in the text. Crucially, the psychometric properties of the MARS scale have been substantiated through multiple studies [30]. It has demonstrated strong objectivity and reliability in an original evaluation study and shown construct and concurrent validity, reinforcing its robustness as a measurement tool.

By utilizing this standardized scale, researchers can ensure a comprehensive, consistent, and objective evaluation of each app. This would allow for a deep understanding of the app's quality and efficacy from multiple perspectives, which would be instrumental in identifying the most appropriate and effective health apps for recommendation or further research.

#### Assessment of mobile applications

To conduct a meaningful assessment of mobile applications, it will be necessary to ensure that evaluators are well-trained and capable of conducting an in-depth analysis. This will likely require the assembly of a diverse team of healthcare professionals to act as evaluators, in line with the scope and target of the apps and the health condition being studied. The composition of the team can vary but it is important to ensure a variety of perspectives and skills. For example, in a previous study [31], to evaluate the characteristics of specific nutritional apps for chronic renal failure available in Italy, we have enrolled 20 healthcare professionals (five dieticians, five nephrologist physicians, and five hemodialysis nurse specialists and five peritoneal dialysis nurses specialists).

Team members should undergo thorough training to familiarize themselves with the specific tool being used for the evaluation, in this case, the MARS scale. If the MARS scale is not available in the native language of the evaluators, training could be conducted via an e-learning course, with training videos and materials in English. However, this is contingent on both the evaluators and the ultimate end-users (patients) having a competent level of understanding of English. It's crucial that all evaluators understand the MARS scale, its sections, and the nuances of its scoring system to ensure a uniform evaluation across different applications. For effective training, evaluators should engage in practical exercises where they test and assess pre-selected apps. The length of testing could vary, but at least 15 min of usage per app should be considered to allow for adequate exploration of its features. As substantiation of this, even in the study conducted by Martinon et all., in order to assess the quality of nutritional mobile applications available in the French market, evaluators downloaded and subjected each application to a minimum testing duration of 15 min [32]. Following this hands-on experience, each evaluator should then complete the MARS scale questionnaire for the application they have tested. Given the subjective nature of some elements of the MARS scale, it is expected that different evaluators might score the same app differently. To ensure more standardized evaluation, some level of consensus should be reached amongst evaluators. For instance, if the scoring difference on any item between evaluators exceeds a pre-set threshold (e.g., two points), evaluators should engage in discussion to better understand each other's views and arrive at a common understanding.

Once training is complete, the assessment process can commence. The length of this process will depend on the number of apps to be evaluated, but sufficient time should be allocated for an in-depth analysis. During this process, each evaluator should independently use each assigned application for an appropriate duration (e.g., 30 min) and then immediately evaluate it using the MARS scale questionnaire. This approach aims to reduce recall bias and ensure that evaluators' impressions of the apps are precise and accurate.

The use of the web-based MARS scale questionnaire would facilitate real-time recording of evaluators' responses, which can be easily collected for subsequent analysis. This procedure ensures a uniform and systematic evaluation process, which is critical to generate reliable and robust data on the quality of the apps under consideration.

**Fig. 1:**
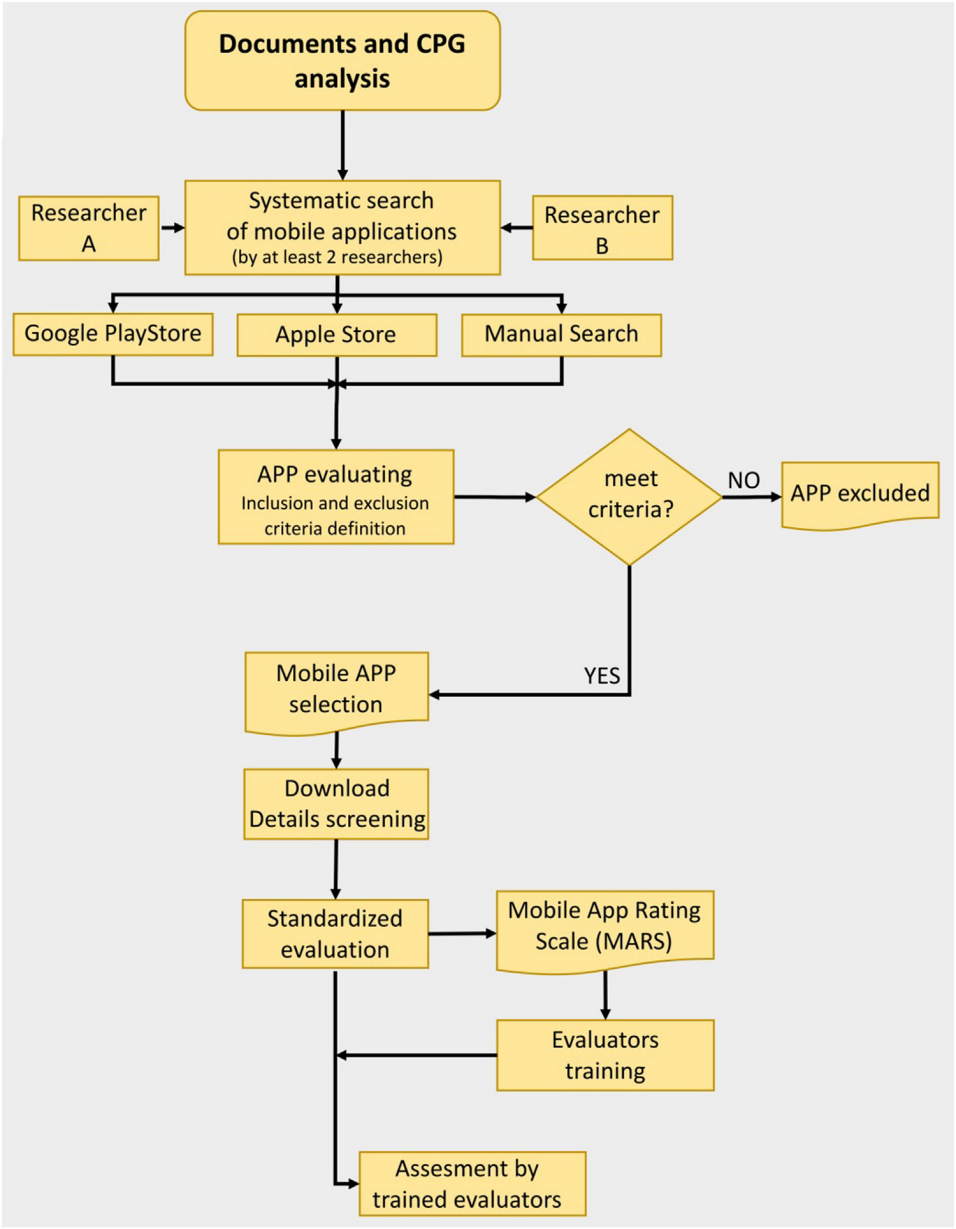
Flowchart of mobile app selection and evaluation process. ***Legend:****APP= Mobile App; CPG= Clinical Practice Guideline; MARS= Mobile App Rating Scale*.

In Fig. 1, a graphical summary of the first phase of the method is shown.

### Data analysis

#### Data synthesis

The selected mobile applications should be organized according to the specific objectives of the investigation; then the findings and comprehensive conclusions should be summarized for each individual objective. These should be reported, as for example in a literature review, both as a narrative summary and as statistical results, in well-structured tables [33].

These tables should provide a comprehensive and transparent summary of the study's findings. For instance, they could include the name of the mobile application, the overall MARS score, as well as the score for each MARS subcategory. This would offer a detailed insight into the strengths and weaknesses of each app, enabling a more direct comparison amongst them. Furthermore, these tables could provide additional details about each app, such as the target population, the health condition addressed, and any other notable features, thereby enriching the depth of information available to the end user.

The presentation of results as a narrative synthesis and in tables facilitates clear communication of complex data. It provides an efficient way to share and interpret the study's findings while also allowing for a more detailed examination of the data where necessary.

#### Statistical analysis

The data collected should be anonymously input into a specialized database and analyzed using an appropriate statistical software package. The specific software chosen should be capable of conducting a wide range of analyses, from descriptive to inferential statistics, and should be well equipped to handle large datasets efficiently. Descriptive statistics, such as the median, the interquartile range (IQR), and frequencies, should be employed to summarize the data. The apps should be evaluated using the MARS questionnaire. For the analysis of ordinal variables, the Mann-Whitney *U* test could be applied. The Mann-Whitney *U* test is a non-parametric test, which signifies that it does not rely on any assumptions about the distribution of the data. It is commonly deployed in cases where the data are not normally distributed or where the sample sizes are small. Two-tailed p-values less than 0.05 should be deemed as statistically significant.

The choice of the statistical test should be guided by the nature of the data, the research question at hand, and the distribution of the data. Emphasis should be placed on ensuring that any statistical analysis adheres to the principles of transparency and rigor, guaranteeing that the results are both reliable and robust. Utilizing these methods will bolster the integrity of the findings and provide more valuable insights from the collected data.

**Fig. 2:**
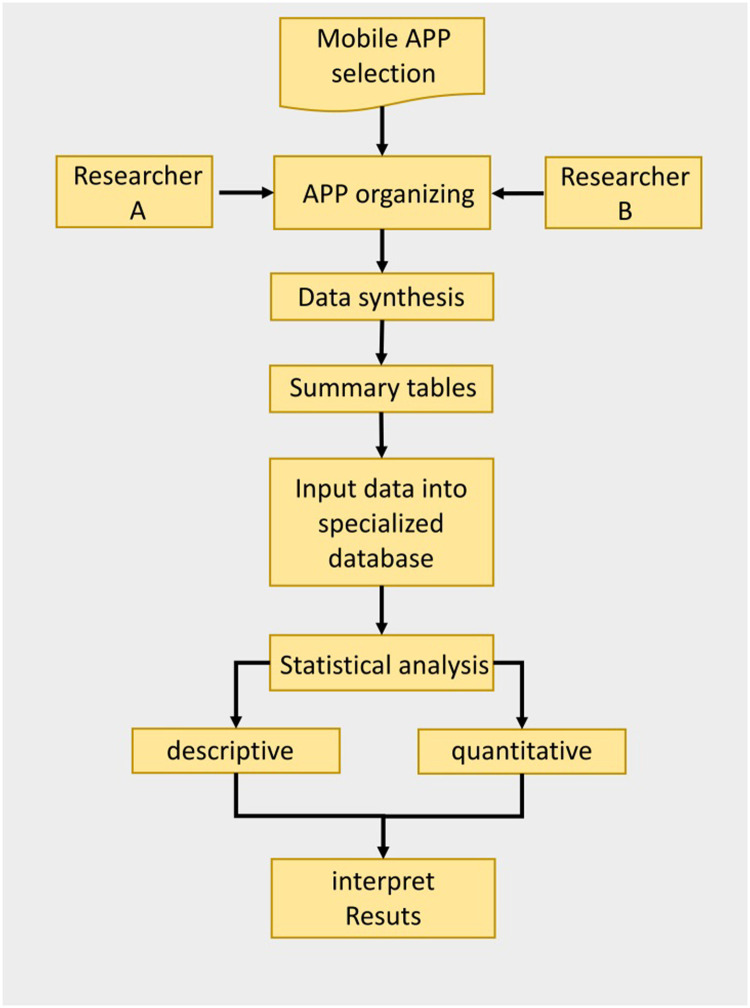
Flowchart of data analysis and synthesis procedure.

In Fig. 2, a graphical summary of the second phase of the methodology is shown.

## Protocol limitations

A possible limitation of this methodology lies in suggesting a strategy for assessing health apps based solely on a limited number of criteria arbitrarily evaluated as important and encompassed within the MARS scale. A future development could involve the validation of an indicator system for assessing the appropriateness of a quality evaluation scale for health apps.

## Conclusions and implications for clinical practice

This method has established a methodology for the assessment of health applications available on the Apple App Store and the Google Play Store with the objective of aiding teams of healthcare professionals or research groups in the selection of apps to be employed for e healthcare consumers use. Particularly, through its proper application, this method can assist healthcare practitioners in discerning which apps to incorporate to bolster clinical practice and which to discard due to their unsuitability concerning the effectiveness of proposed healthcare features, accuracy of information provision, adherence to clinical guidelines, data security, privacy, usability, and accessibility. Indeed, these aspects represent common risks within the realm of mHealth implementation in healthcare.

## Supplementary file

English Version of the Mobile App Rating Scale (MARS) available at: doi:10.2196/mhealth.3422.

## CRediT author statement

MS: Conceptualization, Methodology, Writing Original Draft, Review & Editing, Investigation, Visualization; DA: Conceptualization, Methodology, Writing Original Draft, Review & Editing, Investigation, Visualization; LG: Methodology, Writing Original Draft, Statistical Analysis, Review & Editing, Visualization, Coordinator; DG: Methodology, Writing Original Draft, Review & Editing, Visualization, Coordinator.

MS & DA provided an equal contribution as first author in drafting the manuscript; LG & DG provided an equal contribution to the coordination of the research group. All authors read and approved the final manuscript.

## Declaration of Competing Interest

The authors declare that they have no known competing financial interests or personal relationships that could have appeared to influence the work reported in this paper.

## Data Availability

Data will be made available on request.
